# GPU-Acceleration of Sequence Homology Searches with Database Subsequence Clustering

**DOI:** 10.1371/journal.pone.0157338

**Published:** 2016-08-02

**Authors:** Shuji Suzuki, Masanori Kakuta, Takashi Ishida, Yutaka Akiyama

**Affiliations:** 1 Graduate School of Information Science and Engineering, Tokyo Institute of Technology, Meguro-ku, Tokyo, Japan; 2 Education Academy of Computational Life Sciences (ACLS), Tokyo Institute of Technology, Meguro-ku, Tokyo, Japan; Max F. Perutz Laboratories, AUSTRIA

## Abstract

Sequence homology searches are used in various fields and require large amounts of computation time, especially for metagenomic analysis, owing to the large number of queries and the database size. To accelerate computing analyses, graphics processing units (GPUs) are widely used as a low-cost, high-performance computing platform. Therefore, we mapped the time-consuming steps involved in GHOSTZ, which is a state-of-the-art homology search algorithm for protein sequences, onto a GPU and implemented it as GHOSTZ-GPU. In addition, we optimized memory access for GPU calculations and for communication between the CPU and GPU. As per results of the evaluation test involving metagenomic data, GHOSTZ-GPU with 12 CPU threads and 1 GPU was approximately 3.0- to 4.1-fold faster than GHOSTZ with 12 CPU threads. Moreover, GHOSTZ-GPU with 12 CPU threads and 3 GPUs was approximately 5.8- to 7.7-fold faster than GHOSTZ with 12 CPU threads.

## Introduction

Sequence homology search is widely used in bioinformatics. This method is needed to identify evolutionary relations among sequences. It can also be used to estimate possible functions and structures of DNA and proteins. Nonetheless, sequence homology searches have become a major bottleneck of such analyses, especially metagenomic analyses, because of the increasing number of queries and database size.

In a metagenomic analysis, environmental samples frequently include DNA sequences from several species, and the reference database often does not contain closely related homologous sequences. Therefore, a sequence homology search is used to identify novel genes in these samples. In a typical metagenomic analysis, reads are translated into protein-coding sequences and assigned to protein families by means of a homology search in publicly available databases such as the Kyoto Encyclopedia of Genes and Genomes (KEGG) [1, 2], COG [3, 4] and Pfam [5]. The BLASTX software [6, 7] is commonly used for such binning and classification searches. To identify homologs that may not have high identity of nucleotide sequences, BLASTX translates nucleotide sequences into protein sequences because such sequences are often more similar than the original nucleotide sequences [8, 9]. Nonetheless, the search speed of BLASTX is insufficient for analysis of large quantities of sequence data now available. For instance, sequence homology searches for metagenomic data produced by HiSeq2500, which is one of the latest DNA sequencers, require approximately 100,000 days with BLASTX on a single workstation containing 12 CPU cores.

Developers of several homology search tools currently available, such as RAPSearch [10, 11], GHOSTX [12], GHOSTZ [13], and DIAMOND[14], claim that their tools search faster than BLASTX, without a significant decrease in search sensitivity. GHOSTZ is one of the fastest homology search tools. It uses the database subsequence clustering method. This method clusters similar subsequences from a database to perform an efficient seed search and ungapped extension by reducing the number of alignment candidates on the basis of triangle inequality. GHOSTZ achieved a 2-fold increase in speed, without a substantial decrease in search sensitivity, as compared to GHOSTZ without the database subsequence clustering method. Originally, GHOSTZ was approximately 2.2–2.8 times faster than RAPSearch and approximately 185–261 times faster than BLASTX. Nevertheless, the sequencing technology has since improved and bigger sequence data can now be obtained. Therefore, the speed of homology searches needs a further increase to facilitate efficient metagenomic analysis.

To accelerate computing analyses, graphics processing units (GPUs) are widely used as a low-cost high-performance computing platform. Among top-level supercomputers worldwide, several systems incorporate multiple CPU cores and GPUs within a node, as in TSUBAME 2.5 of the Tokyo Institute of Technology. GPUs have greater computational power and memory bandwidth than CPUs do. Recently, several bioinformatic tools have been enhanced by means of GPUs [15–17].

Several tools for sequence homology searches on the basis of GPUs have also been developed. They are roughly classified into 2 types: implementation of the Smith-Waterman algorithm [18] and a seed-and-extend algorithm such as BLAST. GPU accelerated Smith-Waterman algorithms [19–21] and seed-and-extend algorithms for DNA sequences [22, 23] were several times faster than those of implementation for CPU with multiple CPU cores. SW#db [20] is one of the GPU-based Smith-Waterman algorithms, and showed that when it is based on 1 GPU, it works 4- to 5-fold faster than does SSEARCH [24], which is a CPU-based Smith-Waterman algorithm, with 4 CPU cores. G-BLASTN [22] is one of the GPU-based BLAST with 1 GPU achieves 7.2-fold acceleration relative to the MEGABLAST mode of NCBI-BLAST [25] with 4 CPU cores and 1.6-fold acceleration relative to the BLASTN mode of NCBI-BLAST with 4 CPU cores. On the other hand, GPU accelerated seed-and-extend algorithms for protein sequences, such as GPU-BLAST [26] and CUDA-BLASTP[27], achieved limited success. GPU-BLAST [26] and CUDA-BLASTP[27] with 1 GPU achieve 6-fold and 5- to 6-fold acceleration, respectively, relative to the BLASTP mode of NCBI-BLAST with a single CPU core. These results mean that the acceleration of GPU-BLAST or CUDA-BLASTP with 1 GPU is estimated to be less than 1.5-fold as compared to NCBI-BLAST with a single CPU socket with 4 CPU cores. Therefore, mapping an algorithm of a protein sequence homology search onto GPUs is still a challenging task. In addition, faster algorithms of protein sequence homology searches than BLAST have not yet been mapped onto GPUs.

In this study, we mapped the GHOSTZ algorithm onto GPUs to accelerate sequence homology searches. This task was more challenging than GPU implementation of BLASTX because GHOSTZ is faster than BLASTX. We introduced several speed-up methods in addition to simple GPU mapping of the algorithm. To accelerate a sequence homology search on GPUs, we optimized access of the data in the GPU memory. Moreover, we reduced the waiting time for synchronization to attain full use of a computing environment by setting up GHOSTZ-GPU to reduce inactive threads in gapped extension and to use asynchronous execution on CPU and GPU. GHOSTZ-GPU was implemented in C++ and CUDA 6.0. It is distributed under the BSD 2-clause license and is available for download at https://github.com/akiyamalab/ghostz-gpu.

## Methods

### GHOSTZ

**Fig 1:**
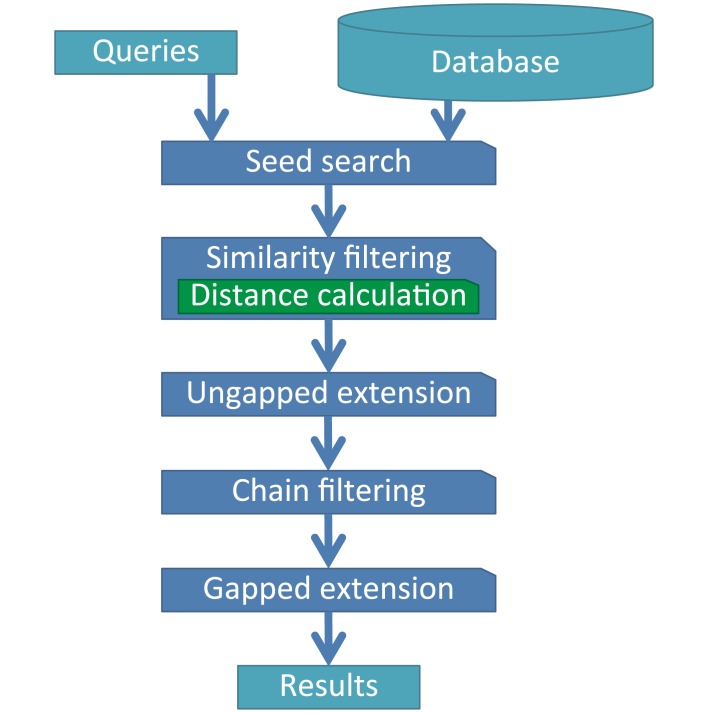
The workflow of GHOSTZ.

The workflow of GHOSTZ is shown in Fig 1. The GHOSTZ protocol consists of 5 main steps: seed search, similarity filtering, ungapped extension, chain filtering, and gapped extension. To accelerate the sequence homology search with the GHOSTZ algorithm, subsequences are extracted from database sequences and similar subsequences are clustered during preprocessing for the sequence homology search. Subsequently, hash tables are constructed containing indexes for the subsequences within database sequences. GHOSTZ uses the hash tables to select seeds for the alignments from representative sequences in the clusters. In the sequence homology search, the seed search process selects seeds that are subsequences of database sequences similar to those of the query sequence. Similarity filtering is then performed to reduce the number of seeds, whereby the distance between a query subsequence and the cluster representative is calculated, and the lower bounds of the distance between the query subsequence and other members of the cluster are computed on the basis of triangle inequality. If the computed lower bound is lower than or equal to the distance threshold, then the seed is taken to the next step, that is, the ungapped extension, to assess the homology between the query and the member sequences of the cluster. Finally, chain filtering is used to bring similar extended seeds together, and gapped extension is performed to obtain an alignment from the extended seed that contains gaps.

The GHOSTZ system has several limitations. Table 1 shows the ratio of calculation time for each step of GHOSTZ. The ungapped-extension step requires most (44.3%) of the total calculation time. Nonetheless, mapping of an ungapped extension onto GPUs by itself is insufficient for improvement of the search speed. This is because other calculations also require considerable time, for example, gapped extension and the distance calculation, which is a subprocess of similarity filtering.

**Table 1:** Average and standard deviation of computation time of each component of a GHOSTZ calculation with 1 CPU thread.

	CPU Time (sec.)	Ratio (%)
Distance calculation	1340.7 ± 45.7	3.2 ± 0.0
Ungapped Extension	18554.8 ± 695.8	44.3 ± 0.1
Gapped Extension	17191.5 ± 672.7	41.1 ± 0.2
Others	4772.6 ± 116.5	11.4 ± 0.3
Total	41859.6 ± 1499.1	100.0 ± 0.0

This profile was obtained from the calculation involving 1,000,000 short DNA reads in metagenomic sequences of a soil microbiome (accession number SRR407548, read length 150 bp) as queries and KEGG GENES (as of May 2013) as the database. The reads were randomly selected from dataset SRR407548. The profile was obtained on a workstation with a single CPU core of 2.93 GHz (Intel Xeon 5670 processor) and 54 GB of memory. GHOSTZ was compiled by means of GCC (version 4.3.4) with the -O3 optimization option. To obtain a profile, the functions of distance calculation, ungapped extension, and gapped extension were not in-lined. This experiment was repeated 5 times.

### GPU Implementation Overview

To improve the search speed of GHOSTZ with GPUs, the mapping of the steps, including distance calculation, ungapped extension, and gapped extension, onto GPUs is evidently crucial for achievement of effective acceleration. Therefore, we mapped these steps onto GPUs. Current computing systems often have multiple CPU cores and multiple GPUs in a computing node. Therefore, we focused on a computing node with multiple CPU cores and multiple GPUs. For the GPU implementation, we used NVIDIA’s CUDA 6.0.

The CUDA software contains a function (performed on a GPU) called a kernel. It represents the operations to be launched by a single CPU thread and is invoked as a set of concurrently executed GPU threads. These threads are organized in a hierarchy consisting of thread blocks and grids. A thread block is a set of concurrent threads, and a grid is a set of independent thread blocks. The kernel uses several types of memory, such as global memory, local memory, shared memory, and registers. Global memory is used for communication between a CPU and GPU. Local memory stores local variables of a thread when a register is not used. Although global and local memory are larger than other memory types in the GPU, access to them is slow. Therefore, it is important for GPU calculations to reduce the number of accesses to these memory types, often via the use of shared memory, to which the access is faster than to either global or local memory. Shared memory is also used to communicate among threads in a block. However, it is smaller than either global or local memory. Therefore, it is used as a software cache.

Simply mapping the distance calculation, ungapped extension, and gapped extension onto a GPU is insufficient for acceleration of GHOSTZ. It involves a number of accesses to global memory, the large inactive threads in a GPU, and the lengthy computation time of other CPU calculations. Therefore, we applied 4 main optimizations: memory access for sequence data, memory access for dynamic programing (DP) matrices and load balancing in gapped extension, asynchronous execution on the CPU and GPU, and addition of a special thread for loading the database.

### Optimization of the Distance Calculation

The distance calculation is a part of similarity filtering. Distance calculations are independent of each other. Therefore, these calculations can be performed within different GPU threads. Nevertheless, when each thread executes a different task in a block, the memory access for the query or database sequence is random. These memory accesses take a long computational time. Therefore, it is important to utilize efficiency of the GPU to reduce the number of random accesses to global memory for sequence data. We use 2 approaches to reduce the number of random accesses: “vectorized memory access” and “group memory access”.

A character in a protein sequence is represented by 5 bits in GHOSTZ-GPU because the alphabet size for a protein is 20. Therefore, an 8-bit memory module is sufficient for each character in the sequence. On the other hand, if 8 bits are used for character access, then a large number of accesses to global memory are required for the protein sequence. To solve this problem, vectorized load instruction (64- or 128-bit access) is often used to reduce the number of global memory accesses and is called “vectorized memory access” in CUDA programming. When we use this memory access method, the accessed data have to be assigned to a consecutive region in global memory. Suppose *w* is the number of characters to be accessed once and *l* is the length of the sequence for calculation. With this memory access, the number of accesses to global memory is ⌈(*l* + *w* − 1)/*w*⌉. In GHOSTZ-GPU, the sequence data are allocated to consecutive regions in global memory. The sequences in a database are connected with inserted delimiters to transform them into a long single sequence. Query sequences are connected in the same manner as database sequences are. Therefore, GHOSTZ-GPU can use vectorized memory access for sequence data. GHOSTZ-GPU uses 64-bit access to protein sequences. In distance calculations, the length of the sequence used *l* is 10. In the GPU, the bit size of a character is 5. Therefore, when GHSOTZ-GPU uses 64-bit memory access, *w* is 12. In this case, the number of memory accesses for sequence data is 2.

**Fig 2:**
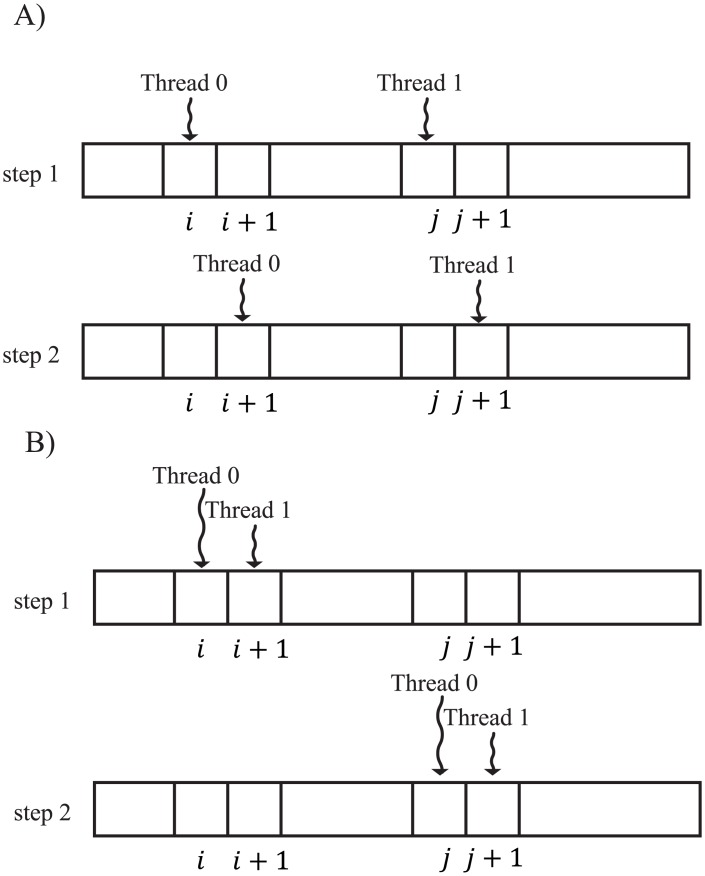
Examples of sequence data accesses. A) An example of sequence data accesses without group memory access. B) An example of sequence data accesses with group memory access.

Moreover, we propose “group memory access” to speed up global memory access. For group memory access, we subdivided threads in a block into small groups. Then, the threads in a group load the data with communication among them. The threads in a group access one consecutive region at a time in global memory. These memory accesses are merged into a single transaction in a GPU, called coalesced memory access, which is used when threads in a block access the same region in global memory. With coalesced memory access for sequence data, the number of memory accesses for sequence data is reduced. The threads that have the same group ID are combined into 1 group. The group ID is calculated as follows. If *i*_*thread*_ is the thread ID and *N*_*member*_ is the number of threads in a group, then let us assume that *i*_*group*_ = ⌊*i*_*thread*_/*N*_*member*_⌋ is the group ID of the thread *i*_*thread*_. Examples of memory access with and without group memory access are shown in Fig 2. For group memory access, we used shared memory to temporarily store sequence data and for communication among threads in a group. The number of accesses to global memory decreases with the use of this memory. In the distance calculation, the number of threads in a group *N*_*member*_ is 2. *l* and *w* have the same value as with vectorized memory access. In this case, the minimal number of memory accesses with group memory access is only 1.

Moreover, the distance calculation also has memory access to global memory for the positions of seeds. In addition, the memory access for the postion of a seed is required for coalesced memory access to reduce the number of global memory accesses. The positions of a seed are a query position and database position. We use the structure of array for the positions of seeds to use coalesced memory access for these data. The structure of an array is often used for coalesced memory access in the structure of GPU computing.

**Fig 3:**
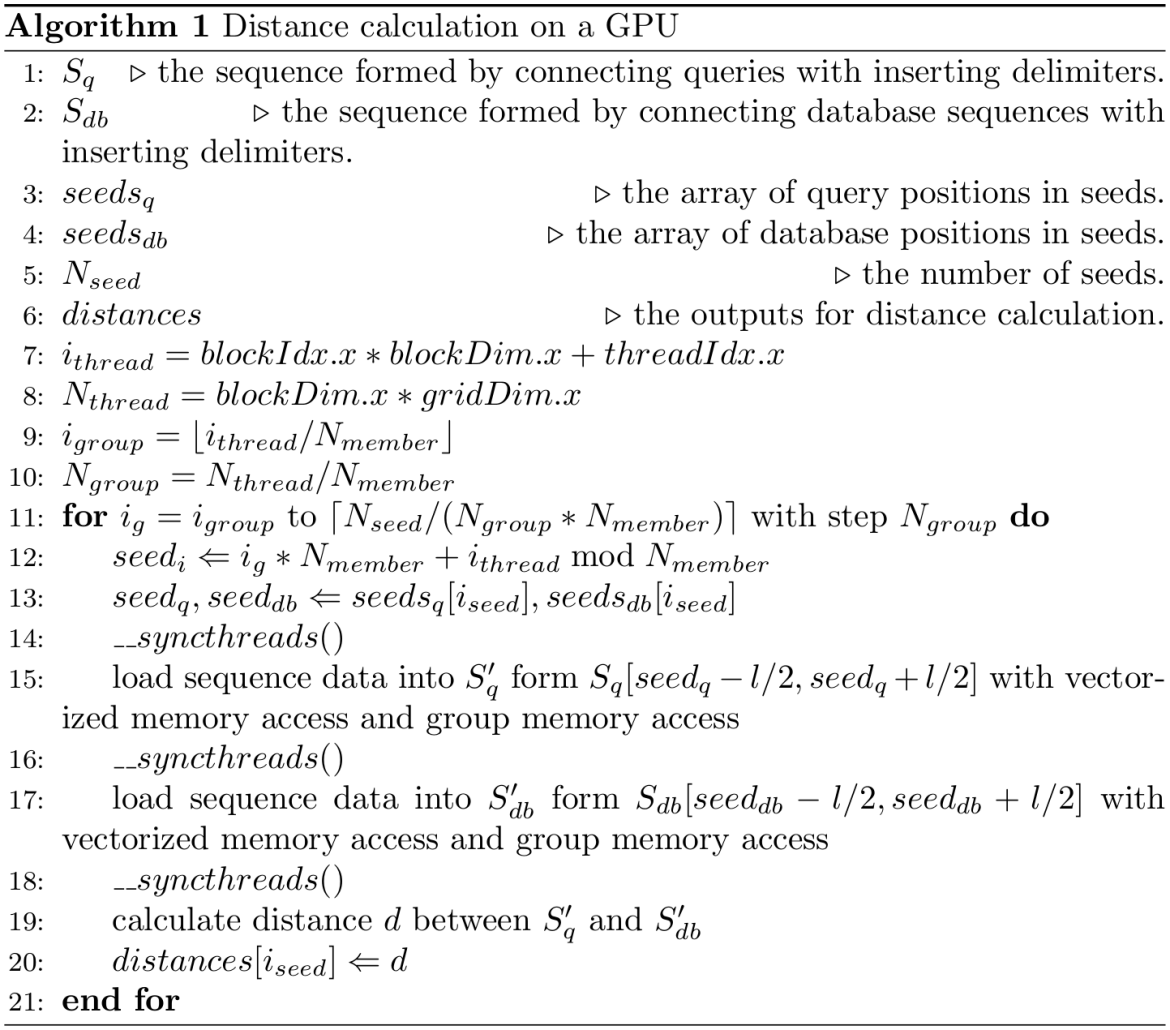
The pseudocode of distance calculation.

Fig 3 shows a pseudocode for distance calculation. The subsequences are loaded into shared memory from global memory with vectorized memory access and group memory access (lines 14 and 15 in Fig 3). After that, the distance between the query subsequence and database subsequence is calculated.

Using these optimizations, we reduced the number of global memory accesses.

### Optimization of Ungapped Extension

Most of homology search algorithms perform ungapped extension before gapped extension to reduce the number of candidates because gapped extension generally requires a lot of computation time. Because ungapped extensions are independent of each other, these calculations can also be performed in different GPU threads.

Ungapped extension requires a number of memory accesses for sequence data and the positions of seeds. We use vectorized memory access, group memory access, and the structure of an array for the positions of seeds during ungapped extension, as in distance calculation. On the other hand, the use of group memory access to all sequence data leads to performance degradation because the lengths of extensions vary (X-dropoff [6, 7] is used [13] for extensions). The threads that finish ungapped extensions wait for the other threads. Thus, we use group memory access only for the first memory access for each sequence. The number of members in a group is 4. If more memory accesses are required, GHOSTZ-GPU uses only vectorized memory access for the remaining sequence data.

**Fig 4:**
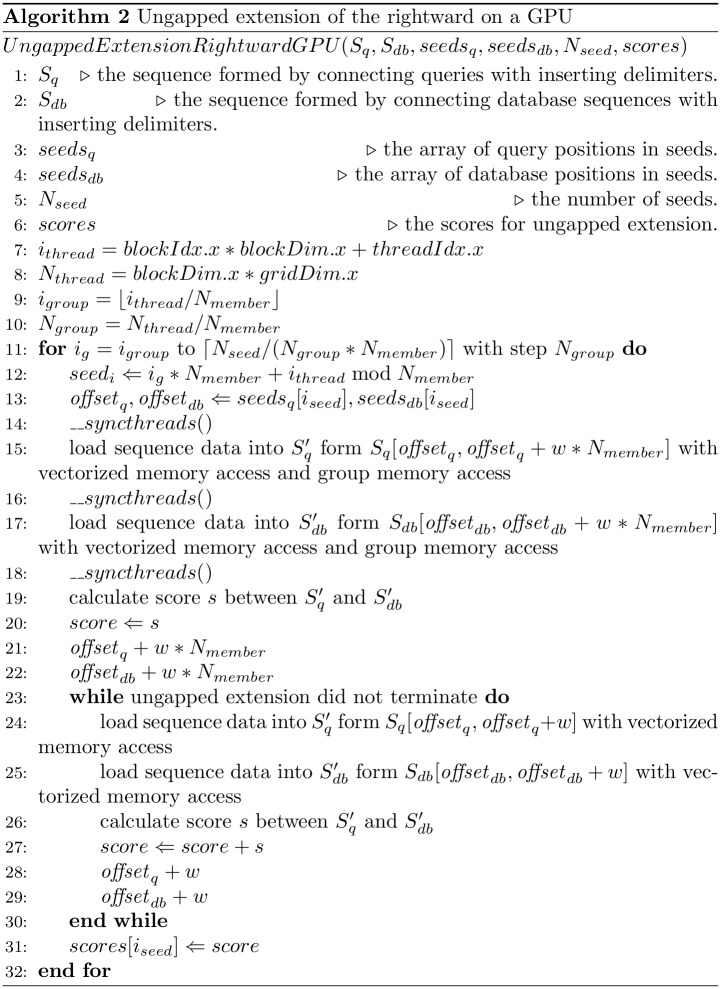
The pseudocode of ungapped extension.

Fig 4 shows a pseudocode for ungapped extension of the rightward on a GPU. Ungapped extension of the leftward on a GPU is almost the same as ungapped extension of the rightward on a GPU. The subsequences are loaded into shared memory from global memory with vectorized memory access and group memory access (lines 15 and 16 in Fig 4). Then, the ungapped-extension score between the query subsequence and database subsequence is calculated. If the ungapped extension is not terminated in line 17 of Fig 4, then the ungapped extension is continued until its termination (lines 23–30 in Fig 4). The lengths of this loop are different for every seed. Thus, group memory access is not used in this loop (lines 24 and 25 in Fig 4).

### Optimization of Gapped Extension

During gapped extension, the seeds are extended with gaps. Gapped extensions are independent of each other. Therefore, these calculations can be performed in different GPU threads. There are 2 main reasons why gapped extension increases computation time in a GPU calculation: the access to global memory and branch divergence.

**Fig 5:**
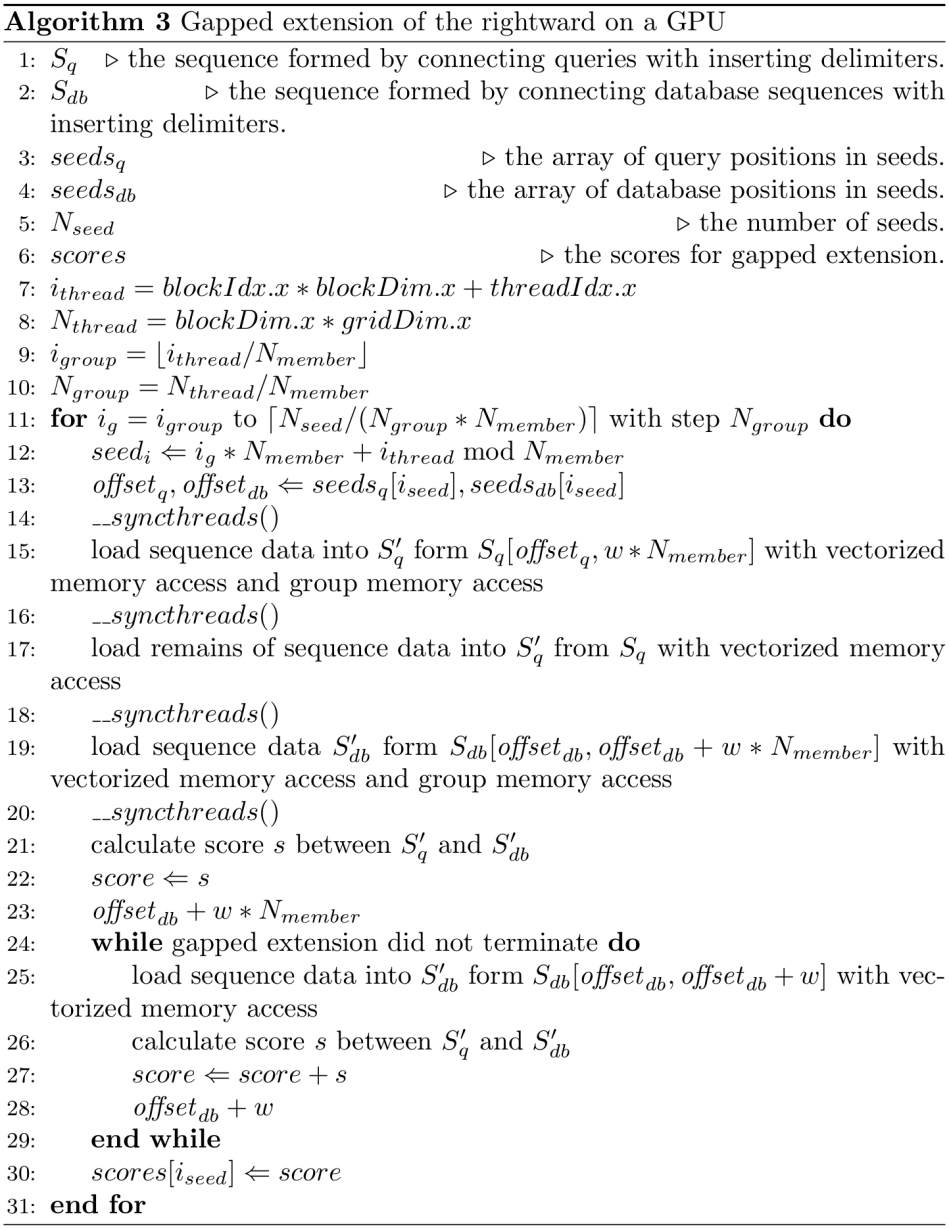
The pseudocode of gapped extension.

Gapped extension mainly involves memory accesses for sequence data, the positions of seeds, and the DP matrix. Memory accesses of gapped extensions for sequence data and the positions of seeds are also optimized in the same manner as for ungapped extensions. Fig 5 shows a pseudocode for gapped extension of the rightward on a GPU. Gapped extension of the leftward on a GPU is almost the same as gapped extension of the rightward on the GPU. The subsequences are loaded from global memory with vectorized memory access and group memory access (lines 15 and 19 in Fig 5). The remaining sequence data are loaded with vectorized memory access (lines 17 and 25 in Fig 5). After loading the sequence data, we calculate the score from these sequence data. If the gapped extension is not terminated in line 21 of Fig 5, then the gapped extension is continued until the termination (lines 24–29 in Fig 5).

**Fig 6:**
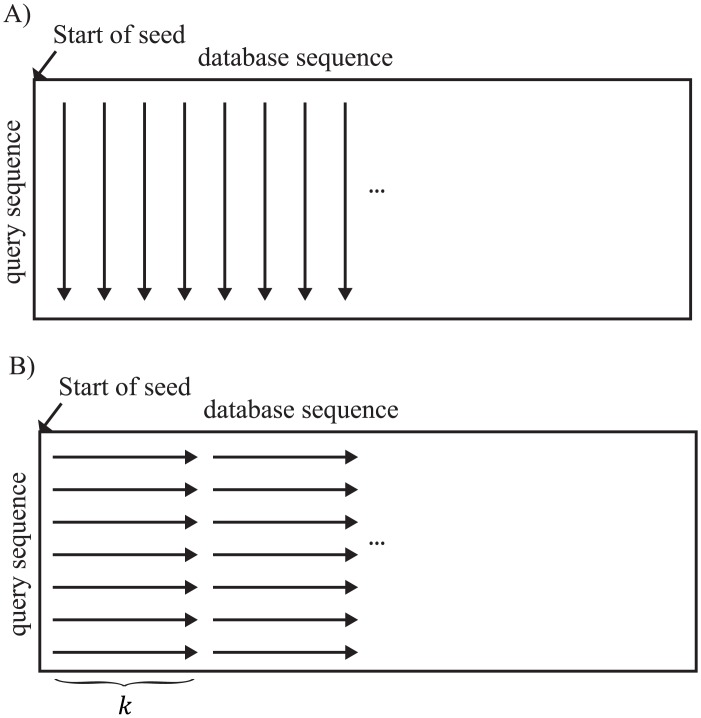
Examples of gapped extension on a GPU. A) An example of accesses for sequence data in GHOSTZ. B) An example of gapped extension with the short loop.

In addition, memory accesses for the DP matrix in gap extensions also require a lengthy computation time. Each cell in the DP matrix is computed by other cells, and the greatest value of a cell is termed “the score” in gapped extension. When we calculate a gapped-alignment score only, we do not need to store all data in the DP matrix. Thus, GHOSTZ-GPU stores only 1 column of the DP matrix in local memory of the GPU in the same way as BLAST does [7]. The length of a column in the DP matrix depends on the query length, which is generally shorter than the database sequence in current metagenomic analyses. Local memory in a GPU is slower than a register or shared memory. For acceleration of the gapped-extension process, we have to reduce the number of accesses to local memory. This task is accomplished by adding another loop to those used to calculate gapped extension and by recruiting shared memory for this loop. The calculation workflows of GHOSTZ and GHOSTZ-GPU during gapped extension are shown in Fig 6. The loop length *k* in GHOSTZ-GPU is 4. This loop requires additional memory access for this loop. Nevertheless, the required data for this loop can be assigned to shared memory. Therefore, the memory accesses for this loop are quick. The shared memory during gapped extension is reused as in group memory access. Thus, the additional shared memory allocations for this loop are not needed. With this optimization, the search results with GHOSTZ-GPU may be different from those with GHOSTZ. The gapped extension is terminated when the score drops by more than *X* below the maximal score previously seen. Thus, the difference in the calculation order for filling the cells in the DP matrix may shift the terminated cells when X-dropoff is used. Nonetheless, the alignments are not changed when they have a high score. This is because the alignment paths rarely pass though the cell near a terminated one. Therefore, we believe that the search results are influenced by this optimization only slightly.

Moreover, it is important to decrease branch divergence to reduce the computation time of gapped extension. Several threads in a block in the GPU execute the same instruction at any given moment, leading to branch divergence. For instance, when some threads in a block run an “if” statement, threads split in two for the branch, and the GPU causes all paths to be executed sequentially, even though each thread executes only one of the paths. On the other hand, when the threads run a “while” statement, the threads wait for execution of another thread to end. Branch divergence causes an increase in computation time and the number of inactive GPU threads. Therefore, there is a need to reduce branch divergence. We used the DP matrix to calculate the score of gapped extension, and the primary cause of the problem was the difference in size of the DP matrix among gapped extensions. The order of calculations for cells in GHOSTZ-GPU gapped extension is shown in Fig 6B. The loop for query length is an inner one during gapped extension. Therefore, the query length has a greater influence on branch divergence than the database sequence length does. For better load balancing, GHOSTZ-GPU sorts seeds by query length and then assigns a seed to a GPU thread successively. With this approach, the lengths of inner loops in gapped extensions are sorted in GHOSTZ-GPU.xtensions are sorted in GHOSTZ-GPU.

### Asynchronous Execution on a CPU and GPU

To make full use of a computing environment with GPUs, an overlap between CPU and GPU calculations is necessary. GHOSTZ-GPU divides a process with a CPU and GPU into 2 main phases. The first phase consists of a seed search and similarity filtering. The second phase consists of chain filtering and gapped extension. CPU threads calculate data independently in each phase. To create an overlap between CPU and GPU calculations, the double-buffering technique is used for CPU and GPU types of memory. Two buffers are used as input and output to store results of the GPU calculations. Because of this method, the waiting time for synchronization of the CPU and GPU is reduced. Moreover, the computation time of memory copying between a CPU and GPU is hidden by the CPU and GPU calculations.

**Fig 7:**
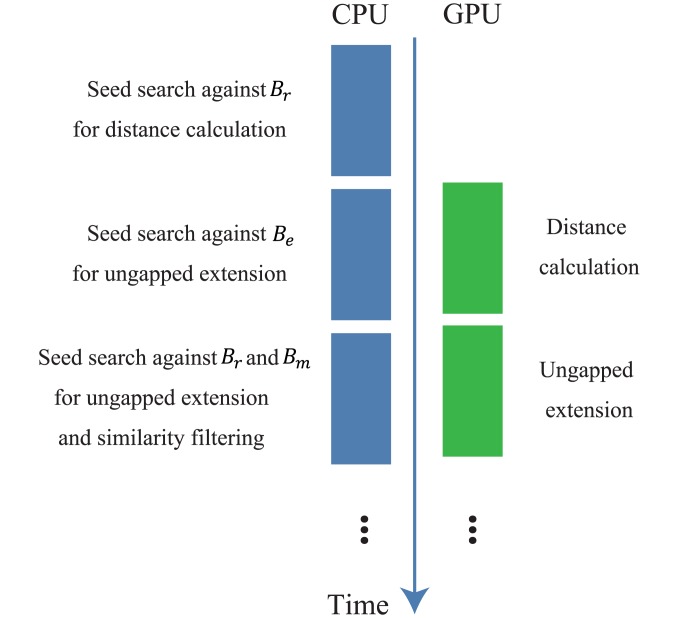
The workflow of the first phase in GHOSTZ-GPU.

The first phase is shown in Fig 7. GHOSTZ uses 3 tables, *B*_*e*_, *B*_*r*_, and *B*_*m*_ for a seed search. *B*_*e*_ is a hash table for the representatives of clusters where the cluster contains only 1 member. *B*_*r*_ is a hash table for a representative of a cluster (not stored in *B*_*e*_). *B*_*m*_ is a table for members of clusters. As shown in Fig 7, a seed search is performed against *B*_*r*_ for distance calculation. Then, distances for similarity filtering are calculated on the GPU. The seed search against hash table is performed on the CPU simultaneously with this GPU calculation because this seed search is independent of similarity filtering. If the distance calculation is finished on the GPU, ungapped-extension calculation on the GPU is initiated. Once the seed search against *B*_*e*_ on the CPU is completed, the seed search and similarity filtering for hash tables *B*_*r*_ and *B*_*m*_ are performed on the CPU. After that, seeds from tables *B*_*r*_ and *B*_*m*_ are built, and ungapped extension for these seeds is performed. This phase is continued until the process for all subsequences of queries is completed.

**Fig 8:**
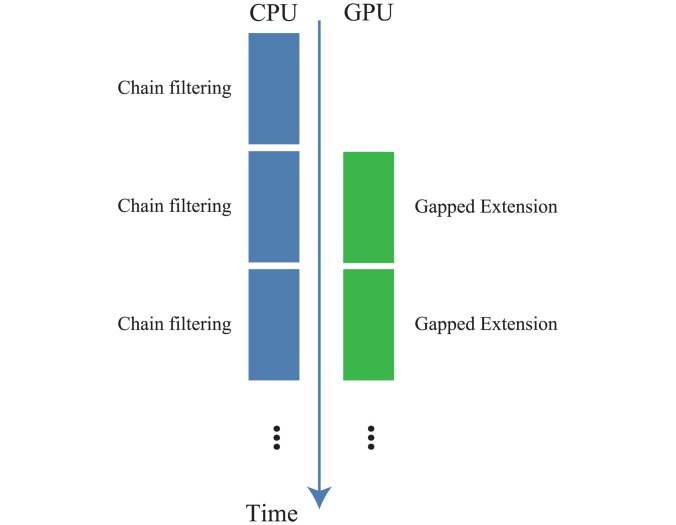
The workflow of the second phase of GHOSTZ-GPU.

The second phase is shown in Fig 8. Chain filtering is performed on the CPU. If the memory is full, then seeds are sorted by query length for alignment, and then gapped extension is performed using these seeds. This phase continues until the process for all seeds is completed.

If multiple GPUs are used, then they are individually used by CPU threads. Each GPU is assigned to almost the same number of CPU threads. Each CPU thread has different global memory in the GPU. Because GPUs do not require communication among one another with this approach, GHOSTZ-GPU utilizes multiple GPUs effectively.

### Optimization of Loading a Database

**Fig 9:**
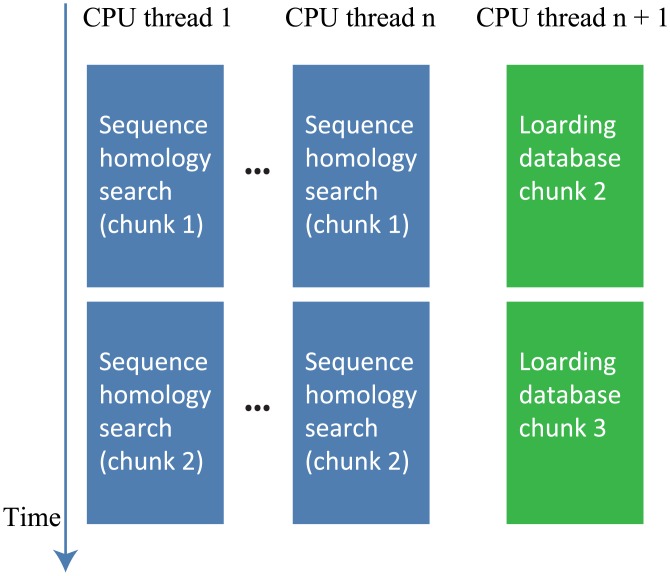
The workflow for loading of a database thread.

Loading a database, including indexes, represents a larger fraction of the computation time in GHOSTZ-GPU than in GHOSTZ because a sequence homology search is faster with a GPU calculation. GHOSTZ-GPU uses a special CPU thread to hide the latency of loading the database from threads for a sequence homology search. In GHOSTZ and GHOSTZ-GPU, a database is divided into several chunks to reduce working memory. The default chunk size is 1 GB. These tools sequentially search each database chunk and merge its results with the results of previous chunk searches. In GHOSTZ-GPU, the chunks are loaded sequentially by a special thread. Fig 9 shows the workflow with this thread. While the other threads perform the sequence homology searches against a database chunk, this thread loads the next database chunk. Due to this approach, the computation time of loading the database is hidden during the sequence homology search.

## Results

### Datasets and Computing Environment

We used the same dataset as Suzuki *et al.*[13] did for evaluation. We used amino acid sequences from the KEGG GENES database (as of May 2013). This database contains approximately 10 million protein sequences, which comprise a total of approximately 3.6 billion residues. For the query sequences, we used 3 datasets: metagenomic sequences of a soil microbiome (accession number SRR407548, read length 150 bp), metagenomic sequences of a human microbiome (accession number SRS011098, read length 101 bp), and metagenomic sequences of a marine microbiome (accession number ERR315856, read length 104 bp). SRR407548 and ERR315856 were obtained from the DNA Data Bank of Japan (DDBJ) Sequence Read Archive. SRS011098 was obtained from the web site of the Data Analysis and Coordination Center for the Human Microbiome Project (http://www.hmpdacc.org/). We used the whole metagenomic shotgun sequencing data from dataset SRS011098. To evaluate the computation time, 1,000,000 randomly selected short DNA reads were used for all datasets. Each experiment was repeated 5 times, but we used only 10,000 randomly selected short DNA reads and used each tool once to evaluate the search sensitivity levels because of the computational costs. All the calculations were conducted on the TSUBAME 2.5 supercomputing system, Tokyo Institute of Technology, Japan. We used this node in all experiments, which consists of two 2.93-GHz Intel Xeon 5670 processors (6 cores), 54-GB memory, 3 NVIDIA Tesla K20Xes, and SUSE Linux Enterprise Server 11 Service Pack 3.

The parameters of GHOSTZ and GHOSTZ-GPU were set to default values. To execute GHOSTZ and GHOSTZ-GPU, similar sequences were arranged close to each other in the database file based on the results of CD-HIT [28] before construction of database indexes.

### Evaluation of the Acceleration by GPUs

**Fig 10:**
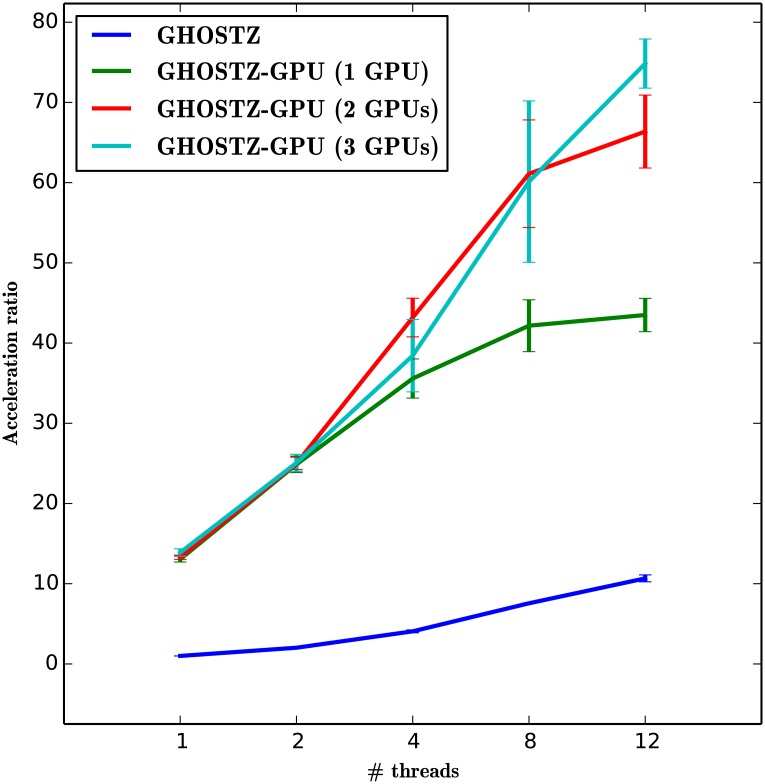
Computation time with multithreading of a CPU and multiple GPUs.

To evaluate acceleration by GPUs and the relation between the number of GPUs and the acceleration, we ran GHOSTZ-GPU and GHOSTZ with their default options, except for the multithreading option. We used 1,000,000 randomly selected short DNA reads from dataset SRR407548 as queries in this evaluation. Fig 10 shows the averages and standard deviations of the computation time for each program with 1, 2, 4, 8, or 12 CPU threads and 1, 2, or 3 GPUs. According to the figure, GHOSTZ and GHOSTZ-GPU with 12 CPU threads show the best performance. In addition, GHOSTZ-GPU showed acceleration of approximately 4.1, 6.2, and 7.7-fold when we used 12 CPU threads with 1, 2, or 3 GPUs, respectively, as compared to GHOSTZ with 12 CPU threads.

### Evaluation of the Acceleration of Each Component by a GPU

For this purpose, we ran GHOSTZ-GPU and GHOSTZ with their default options. We used the same dataset as in Table 1. Table 2 shows the averages and standard deviations of computation time for each step of GHOSTZ with 1 CPU thread and GHOSTZ-GPU with 1 CPU thread and 1 GPU. With a GPU, we found that distance calculation, ungapped extension, and gapped extension in GHOSTZ-GPU were accelerated by the factor of 28.7, 33.3, or 36.6, respectively, relative to GHOSTZ.

**Table 2:** Averages and standard deviations of computation time for each step of GHOSTZ-GPU and GHOSTZ calculations.

	GHOSTZ (sec.)	GHOSTZ-GPU (sec.)	Acceleration Ratio
Distance calculation	1340.7 ± 45.7	46.8 ± 0.1	28.7 ± 1.0
Ungapped Extension	18554.8 ± 695.8	557.5 ± 4.6	33.3 ± 1.4
Gapped Extension	17191.5 ± 672.7	469.1 ± 0.4	36.6 ± 1.4

This profile was obtained from the calculation involving short DNA reads in metagenomic sequences of the soil microbiome (SRR407548) as queries and KEGG GENES as a database. To obtain a profile for GHOSTZ, the functions of distance calculation, ungapped extension, and gapped extension were not in-lined. To obtain a profile for GHOSTZ-GPU, the computation time of memory copy between CPU and GPU was excluded. This is because this process is hidden by CPU and GPU calculations during asynchronous execution on a CPU and GPU.

### Evaluation of Search Sensitivity

To evaluate the search sensitivity of GHOSTZ-GPU, we ran GHOSTZ-GPU, GHOSTZ, RAPSearch (version 2.12), and DIAMOND (version 0.7.9). The sensitivity of the homology search for the different query sequences was estimated using the search results obtained by the Smith-Waterman local alignment algorithm implemented in SSEARCH [24] as the correct results. Because the Smith-Waterman algorithm is based on the dynamic programming algorithm and does not use any heuristics, it returns an optimal local alignment. The performance was estimated in terms of the fraction of the results that corresponded to the correct result. A search result was considered correct when the subject sequence with the highest score in SSEARCH was the same as the subject sequence obtained by each tool. We used only 10,000 randomly selected short DNA reads from SRR407548, SRS011098, and ERR315856 as queries and used each tool once during this evaluation for comparison with the results of SSEARCH. This is because the latter requires a lengthy computational time for large query datasets. To evaluate the software, we executed the RAPSearch program with 2 cases. One involved the default options, and the other involved command line options “-a T”, which instructed the program to perform a fast search [we called it RAPSearch (fast)]. We executed the DIAMOND program with 2 cases. One involved the “-c 1” [we called it DIAMOND (fast)], and the other involved command line options “-c 1 --sensitive” [we called it DIAMOND (sensitive)]. “-c” instructed the program to change the number of chunks for processing the seed index. “--sensitive” instructed the program to perform a sensitive search.

**Fig 11:**
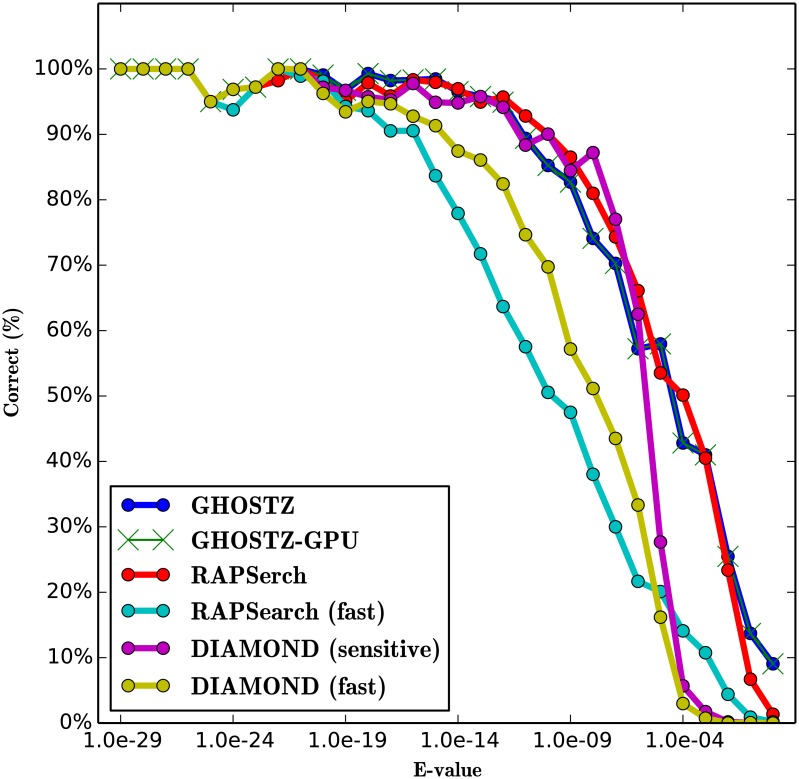
Search sensitivity of different search methods. Searches of SRR407548 sequences against the KEGG GENES database.

**Fig 12:**
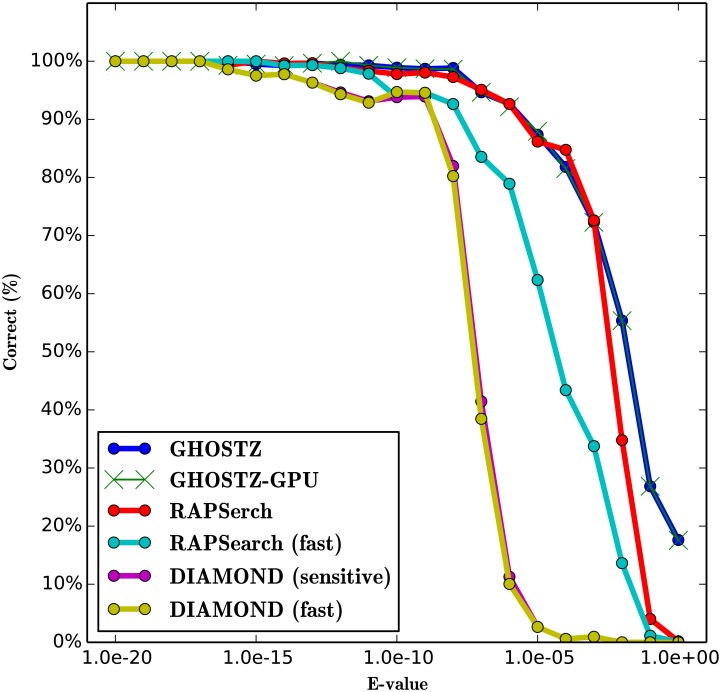
Search sensitivity of different search methods. Searches of SRS011098 sequences against the KEGG GENES database.

**Fig 13:**
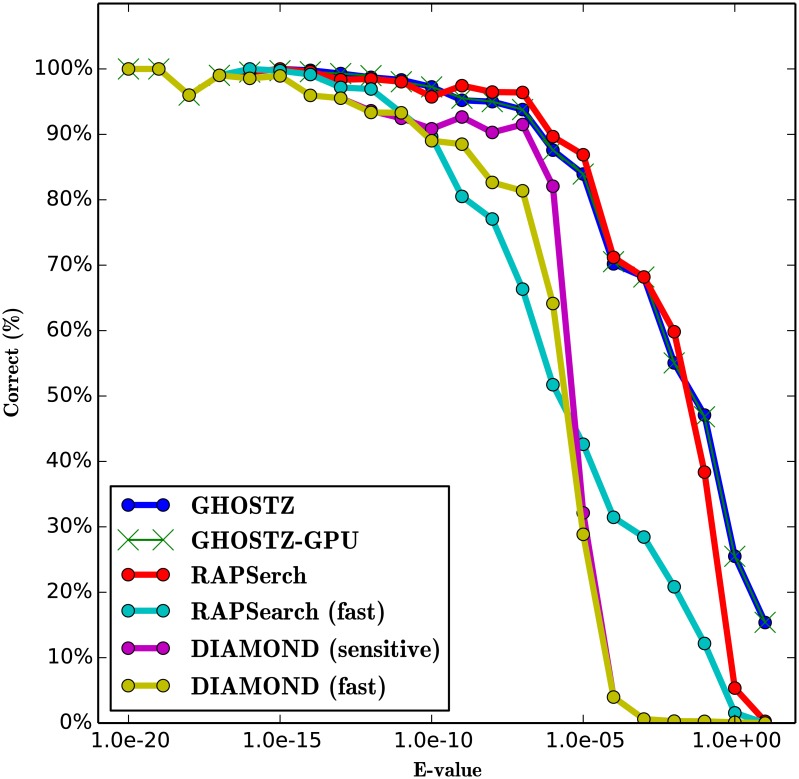
Search sensitivity of different search methods. Searches of ERR315856 sequences against the KEGG GENES database.

The results for SRR407548, SRS011098, and ERR315856 are shown in Figs 11, 12 and 13. These figures indicate that the search sensitivity of GHOSTZ-GPU was almost equal to that of GHOSTZ. Because it is difficult to compare many plots involving the results obtained with different parameters, we used single-value search sensitivity, which is calculated as the ratio of correct queries to all queries whose E-values <1.0 × *E*^−5^ because the hits that have a high E-value are unreliable and not used in practice. For instance, Trunbaugh *et al.* used hits with E values less than 1.0 × *E*^−5^[8], and Kurokawa *et al.* used hits with E values less than 1.0 × *E*^−8^[9]. Table 3 shows search sensitivity for each program. The search sensitivity of GHOSTZ-GPU for SRR407548 was almost equal to that of GHOSTZ, RAPSearch, and DIAMOND (sensitive) and higher than that of RAPSearch (fast) and DIAMOND (fast). The search sensitivity values of GHOSTZ-GPU for SRS011098 and ERR315856 were almost equal to those of GHOSTZ and RAPSearch and higher than those of RAPSearch (fast), DIAMOND (fast), and DIAMOND (sensitive).

**Table 3:** Search sensitivity for SRR407548, SRS011098, and ERR315856.

	SRR407548	SRS011098	ERR315856
GHOSTZ-GPU	0.86	0.98	0.97
GHOSTZ	0.86	0.98	0.97
RAPSearch	0.89	0.98	0.97
RAPSearch (fast)	0.62	0.93	0.88
DIAMOND (fast)	0.72	0.78	0.90
DIAMOND (sensitive)	0.88	0.79	0.93

The search sensitivity is calculated as the ratio of correct queries with the E-values < 10^5^.

Moreover, we compared the subject sequences that had the highest score in the results of GHOSTZ-GPU with those of GHOSTZ. The results of GHOSTZ-GPU were different from those of GHOSTZ. This difference is caused by the difference in the calculation order for filling the cells in the DP matrix during gapped extension. However, the difference between them is only 2–4 queries. Therefore, we believe that GHOSTZ-GPU has sufficient search sensitivity for most of metagenomic applications.

### Evaluation of Computation Time

To further evaluate GHOSTZ-GPU, we compared its computation time with that of GHOSTZ, RAPSearch, and DIAMOND. Optimization of loading the database is also effective for GHOSTZ; therefore, we applied this optimization to GHOSTZ. We evaluated these tools using 1,000,000 randomly selected short DNA reads from datasets SRR407548, SRS011098, and ERR315856 and from the KEGG GENES database. These software packages were used with the same commands that were used to analyze search sensitivity.

The averages and standard deviations of computation time of the tested methods for SRR407548, SRS011098, and ERR315856 are shown in Table 4. GHOSTZ-GPU with 1 GPU was approximately 3.0–4.1, 3.1–3.9, 12.1–19.0, and 0.9–2.0 times faster than GHOSTZ (original), GHOSTZ (+ loading database thread), RAPSearch, and DIAMOND (sensitive) with 12 CPU threads, respectively. Moreover, GHOSTZ-GPU with 3 GPUs was approximately 5.8–7.7, 6.0–7.4, 21.6–35.9, and 1.6–3.8 times faster than GHOSTZ (original), GHOSTZ (+ loading database thread), RAPSearch, and DIAMOND (sensitive) with 12 CPU threads, respectively. GHOSTZ-GPU with 3 GPUs was slower than RAPSearch (fast) with 12 CPU threads on ERR315856 and slower than DIAMOND (fast) with 12 CPU threads on all the data. Nevertheless, the sensitivity of RAPSearch (fast) and DIAMOND (fast) was clearly worse than that of GHOSTZ-GPU. Thus, GHOSTZ-GPU shows the best performance when high sensitivity is required.

**Table 4:** Averages and standard deviations of computation time for datasets SRR407548, SRS011098, and ERR315856.

	SRR407548 (sec.)	SRS011098 (sec.)	ERR315856 (sec.)
GHOSTZ-GPU (1 GPU)	1038.2 ± 47.4	730.8 ± 35.7	1025.3 ± 42.8
GHOSTZ-GPU (2 GPUs)	682.4 ± 56.6	500.9 ± 70.7	701.6 ± 56.6
GHOSTZ-GPU (3 GPUs)	551.5 ± 34.4	375.2 ± 10.2	574.8 ± 17.6
GHOSTZ (+ loading database thread)	4051.6 ± 110.5	2249.8 ± 34.1	3533.8 ± 96.0
GHOSTZ (original)	4231.8 ± 159.7	2178.3 ± 11.0	3560.2 ± 52.1
RAPSearch	19781.2 ± 2349.6	9075.8 ± 101.4	12379.2 ± 192.9
RAPSearch (fast)	996.4 ± 13.2	654.8 ± 5.9	993.1 ± 31.6
DIAMOND (fast)	479.3 ± 55.2	275.6 ± 74.8	279.0 ± 6.7
DIAMOND (sensitive)	2071.7 ± 4.7	713.6 ± 1.9	931.5 ± 18.0

We assessed each tool with 12 CPU threads.

### Evaluation of Optimizations for GPU calculations

To further evaluate GHOSTZ-GPU, we evaluated key optimizations: asynchronous execution on a CPU and GPU, addition of a thread to loading of a database, group memory access, and load balancing of gapped extension. We performed GHOSTZ with 12 CPU threads and GHOSTZ-GPU with 12 CPU threads and 3 GPUs. We ran GHOSTZ-GPU with and without each optimization. We used 1,000,000 randomly selected short DNA reads from SRR407548 against the KEGG GENES database. The acceleration ratios with these optimizations relative to GHOSTZ without the thread for loading a database are shown in Table 5. Each optimization was found to accelerate GHOSTZ-GPU. Asynchronous execution on a CPU and GPU and addition of a thread to the loading of a database yielded the greatest increase in computation speed. Therefore, these optimizations are important for accelerating a search for protein sequence homology using a GPU.

**Table 5:** Averages and standard deviations of computation time and acceleration ratio for each optimization of GHOSTZ-GPU.

	Computation time (sec.)	Acceleration ratio of each optimization	Cumulative acceleration ratio
GHOSTZ	4051.6 ± 110.5	1.0 ± 0.0	1.0 ± 0.0
+ GPU	993.9 ± 21.5	4.1 ± 0.2	4.1 ± 0.2
+ Asynchronous execution	705.9 ± 38.7	1.4 ± 0.1	5.8 ± 0.4
+ Loading database	655.7 ± 11.9	1.1 ± 0.1	6.2 ± 0.2
+ Group memory access	618.7 ± 45.0	1.1 ± 0.1	6.6 ± 0.5
+ Load balancing	551.5 ± 34.4	1.1 ± 0.1	7.4 ± 0.4

We performed GHOSTZ-GPU with and without optimizations. The acceleration in processing speed is shown as the ratio of the time used for GHOSTZ-GPU with an optimization relative to the time used for GHOSTZ-GPU with previous optimization and GHOSTZ.

## Discussion

In this study, we mapped distance calculation, ungapped extension, and gapped extension of GHOSTZ onto a GPU. GHOSTZ-GPU with 2 GPUs is approximately 6 times faster than GHOSTZ with 2 CPU sockets. The accelerated GPU-BLAST and CUDA-BLASTP with 1 GPU are estimated to be equivalent to twice NCBI-BLAST with a single CPU socket or less [26, 27]. Therefore, GHOSTZ-GPU showed a greater increase in speed than GPU-based BLAST tools. One of the reasons for acceleration of calculations with GPUs is the use of seed search in GHOSTZ. BLAST searches consist of 3 main steps: a seed search, ungapped extension, and gapped extension; the bottleneck in BLAST is the seed search. Therefore, the seed search is mapped onto a GPU in these tools. On the other hand, a seed search in BLAST requires several random memory accesses. Random memory access decreases computing speed on a GPU. Accordingly, this step does not utilize sufficient computing resources of GPUs. A seed search by means of other tools also requires random memory access. In contrast, in GHOSTZ, a seed search does not take much computation time, and GHSOTZ is one of the fastest tools for searches for protein sequence homology. Thus, GHOSTZ-GPU showed a significant increase in speed. If we run a distance calculation, ungapped extension, and gapped extension in GHOSTZ on GPUs, the remaining steps become new bottlenecks. For a CPU calculation in GHOSTZ-GPU, the most time-consuming step is the seed search. Nonetheless, this step overlaps with distance calculation and ungapped extension on GPUs. Therefore, the true computation time of the seed search was hidden by that of distance calculation and of ungapped extension on GPUs. Nevertheless, the file I/O in a database accounts for a greater fraction of the computing time for GHOSTZ-GPU. Therefore, GHOSTZ-GPU should be executed with a large amount of queries concurrently to optimize the performance. On the other hand, a greater amount of memory is required than that for execution of a small number of queries. When we used 1,000,000 randomly selected short DNA reads from SRR407548 and the KEGG GENES database, GHOSTZ-GPU required approximately 50 GB of CPU memory for the homology search proper. Thus, the memory size of current typical computing systems may be insufficient for GHOSTZ-GPU. For instance, a node in Titan, which is a supercomputer at the Oak Ridge National Laboratory, has only 32 GB of memory. Therefore, big query data cannot be analyzed by GHOSTZ-GPU at once on this computing system. On the other hand, computer systems with larger memory, e.g., TSUBAME 2.5, are under development and the memory size is increasing. Therefore, GHOSTZ-GPU should soon be available in common computing environments.

GHOSTZ-GPU is developed for homology search of metagenome short reads, but it would be more valuable if it can be used for general protein sequence homology search. To check the point, we compared the sensitivity of GHOSTZ-GPU, RAPSearch, DIAMOND to BLASTP mode of NCBI-BLAST (2.2.28+) using proteins sequences as queries. We employed a method used in a research by Boratyn et al. [20] to evaluate the performance of remote homologue detection of sequence homology search tools. We used ASTRAL40 subset (version 2.06) [29] of the Structural Classification of Proteins (SCOP) [30] database in this evaluation. S1 Fig shows the curves denoting the number of true positives vs. the number of false positives for each tool. The performances of all metagenome homology search tools, GHOSTZ-GPU, RAPSearch and DIAMOND, are clearly less than that of BLAST in general protein sequence homology search, while the tools are much faster than BLAST and has enough search sensitivity for metagenome short reads. The lower search sensitivity of those tools would mainly come from the longer seed length than BLAST. The results indicate that the search sensitivity of GHOSTZ-GPU is insufficient for remote homologue detection, and thus the use of GHOSTZ-GPU is limited in homology search of general protein sequences.

In summary, we developed a GPU version of GHOSTZ, which is the fastest tool for searches for protein sequence homology. Several calculations, distance calculation, ungapped extension, and gapped extension, are bottlenecks in GHOSTZ. We mapped these processes onto GPUs and optimized memory access in the GPU calculation. GHOSTZ-GPU with 12 CPU threads and 1 GPU retains sufficient search sensitivity for practical analyses and is 3.0–4.1 times faster than GHOSTZ with 12 CPU threads. Moreover, GHOSTZ-GPU with 12 CPU threads and 3 GPUs maintains sufficient search sensitivity for practical analyses and is 5.8–7.7 times faster than GHOSTZ with 12 CPU threads. GHOSTZ-GPU on 12 CPU cores and 3 GPUs is estimated to achieve an 1073- to 2010-fold increase in processing speed relative to BLASTX on 12 CPU cores because GHOSTZ was estimated to be approximately 185- to 261-fold faster than BLASTX. If we use GHOSTZ-GPU to analyze the data produced by HiSeq2500 and stored in the KEGG GENES database and approximately 50–100 nodes on TSUBAME 2.5 in metagenomic analysis, the required time is estimated to be only 1 day. On the basis of these estimates, we could perform metagenomic analysis of all data produced by the latest DNA sequencer in real time. At present, the sequencing technology continues to be improved, and the size of sequence data is on the rise. GHOSTZ-GPU and computers with GPUs could be a suitable alternative.

## Supporting Information

S1 Fig Number of true positives vs number of false positives for different search methods on the ASTRAL. The query set was created by sorting the SCOP domains in a lexicographic order and selecting even numbered sequences without queries that are the sole member of the superfamily in ASTRAL 40. In the evaluation, self-hits were ignored. If a hit of a search belongs to the same SCOP superfamily of the query, it was considered as a true positive. And if a hit belongs to the different SCOP fold of the query, it was considered as a false positive. The gapped extension of GHOSTZ-GPU was performed on CPU because gapped extension of GHOSTZ-GPU is designed for short sequence and the size of GPU memory is insufficient for sequences in ASTRAL 40.
(PDF)
