# Supplementary material for: GPU-Acceleration of Sequence Homology Searches with Database Subsequence Clustering
Source: PLoS One. 2016 Aug 2;11(8):e0157338. doi: 10.1371/journal.pone.0157338 (PMC4970815; doi:10.1371/journal.pone.0157338)
Supplement: S1 Fig — The query set was created by sorting the SCOP domains in a lexicographic order and selecting even numbered sequences without queries that are the sole member of the superfamily in ASTRAL 40. In the evaluation, self-hits were ignored. If a hit of a search belongs to the same SCOP superfamily of the query, it was considered as a true positive. And if a hit belongs to the different SCOP fold of the query, it was considered as a false positive. The gapped extension of GHOSTZ-GPU was performed on CPU because gapped extension of GHOSTZ-GPU is designed for short sequence and the size of GPU memory is insufficient for sequences in ASTRAL 40. (PDF) [file pone.0157338.s001.pdf]

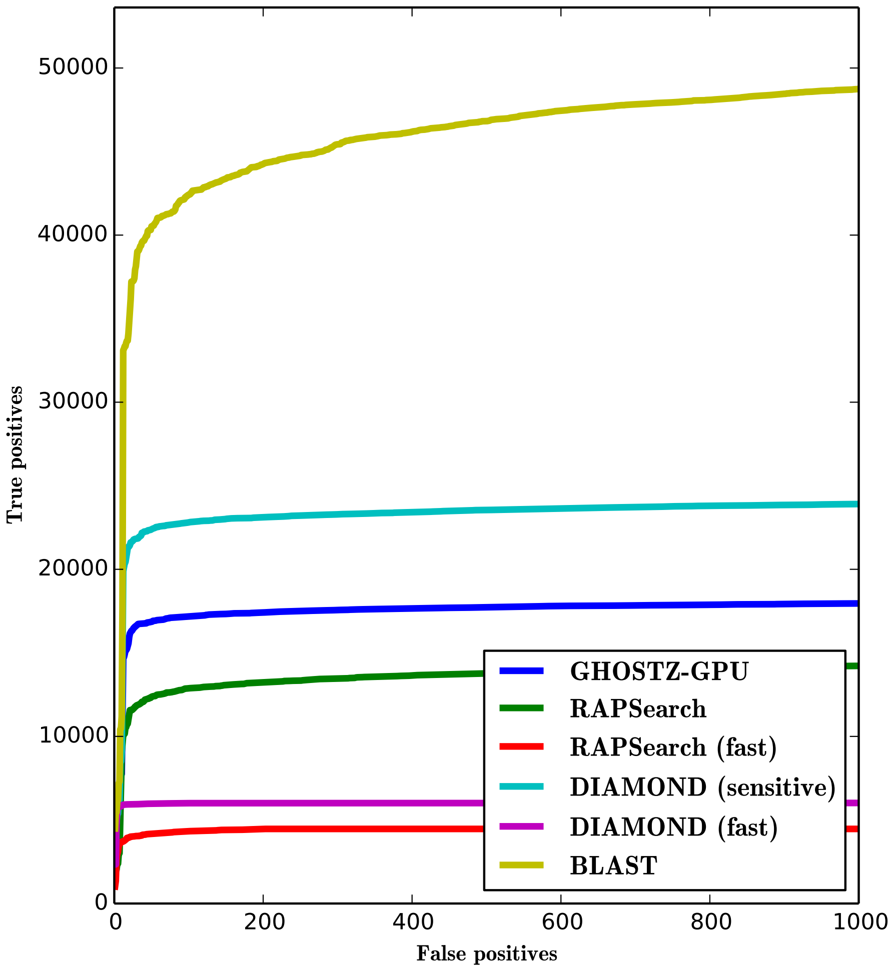

S1 Fig. Number of true positives vs number of false positives for different search methods on the ASTRAL. The query set was created by sorting the SCOP domains in a lexicographic order and selecting even numbered sequences without queries that are the sole member of the superfamily in ASTRAL 40. In the evaluation, self-hits were ignored. If a hit of a search belongs to the same SCOP superfamily of the query, it was considered as a true positive. And if a hit belongs to the different SCOP fold of the query, it was considered as a false positive. The gapped extension of GHOSTZ-GPU was performed on CPU because gapped extension of GHOSTZ-GPU is designed for short sequence and the size of GPU memory is insufficient for sequences in ASTRAL 40.
